# RANKL/OPG ratio regulates odontoclastogenesis in damaged dental pulp

**DOI:** 10.1038/s41598-021-84354-y

**Published:** 2021-02-25

**Authors:** Daisuke Nishida, Atsushi Arai, Lijuan Zhao, Mengyu Yang, Yuko Nakamichi, Kanji Horibe, Akihiro Hosoya, Yasuhiro Kobayashi, Nobuyuki Udagawa, Toshihide Mizoguchi

**Affiliations:** 1 Oral Health Science Center, Tokyo Dental College, Tokyo, 101-0061 Japan; 2 Department of Orthodontics, Matsumoto Dental University, Nagano, 399-0781 Japan; 3 Institute for Oral Science, Matsumoto Dental University, Nagano, 399-0781 Japan; 4 Department of Oral Histology, Matsumoto Dental University, Nagano, 399-0781 Japan; 5 Department of Histology, School of Dentistry, Health Sciences University of Hokkaido, Hokkaido, 061-0293 Japan; 6 Department of Oral Biochemistry, Matsumoto Dental University, Nagano, 399-0781 Japan

**Keywords:** Cell biology, Diseases, Pathogenesis

## Abstract

Bone-resorbing osteoclasts are regulated by the relative ratio of the differentiation factor, receptor activator NF-kappa B ligand (RANKL) and its decoy receptor, osteoprotegerin (OPG). Dental tissue-localized-resorbing cells called odontoclasts have regulatory factors considered as identical to those of osteoclasts; however, it is still unclear whether the RANKL/OPG ratio is a key factor for odontoclast regulation in dental pulp. Here, we showed that odontoclast regulators, macrophage colony-stimulating factor-1, RANKL, and OPG were detectable in mouse pulp of molars, but OPG was dominantly expressed. High OPG expression was expected to have a negative regulatory effect on odontoclastogenesis; however, odontoclasts were not detected in the dental pulp of *OPG-deficient* (*KO*) mice. In contrast, damage induced odontoclast-like cells were seen in wild-type pulp tissues, with their number significantly increased in *OPG-KO* mice. Relative ratio of RANKL/OPG in the damaged pulp was significantly higher than in undamaged control pulp. Pulp damages enhanced hypoxia inducible factor-1α and -2α, reported to increase RANKL or decrease OPG. These results reveal that the relative ratio of RANKL/OPG is significant to pulpal odontoclastogenesis, and that OPG expression is not required for maintenance of pulp homeostasis, but protects pulp from odontoclastogenesis caused by damages.

## Introduction

Osteoclasts are monocyte/macrophage-derived multinucleated bone-resorbing cells^1–3^. Bone tissue is continuously resorbed by osteoclasts, and is subsequently replenished by osteoblasts^4^. Osteoclast differentiation factors, macrophage colony-stimulating factor-1 (CSF-1) and receptor activator of nuclear factor kappa-Β ligand (RANKL), are expressed by bone-forming cells such as osteoblasts and osteocytes^5,6^. Osteoclast precursors express receptors for CSF-1 (CSF-1R) and RANKL (RANK), and differentiate into mature osteoclasts in response to ligand stimulation. In addition, bone-forming cells express OPG, a decoy RANKL receptor that negatively regulates osteoclastogenesis. Therefore, osteoclastic differentiation is tightly regulated by the relative ratio of RANKL and OPG in the tissue microenvironment.

Dental tissue-localized hard tissue-resorbing cells are called odontoclasts. Odontoclasts express some osteoclast markers, such as vacuolar type H^+^-ATPase (V-ATPase), tartrate-resistant acid phosphatase (TRAP), cathepsin K, and matrix metalloprotease-9 (MMP-9), and have structural features similar to osteoclasts^7,8^. Therefore, odontoclasts are considered identical to osteoclasts, the differentiation of which is regulated by CSF-1, RANKL, and OPG. However, the regulation of odontoclastogenesis by these molecules in the dental pulp environment is not completely understood. Bone-resorbing cells in dental tissue are induced less frequently than in bone tissues, and are observed mostly during, inflammation accompanied by deciduous teeth fall out, caries, trauma, and orthodontic movements^9–13^. In addition, pathological odontoclastogenesis induced in the pulp is less frequent than the external resorption occurring on the outside of the tooth^14^. These observations suggest the presence of an odontoclastogenesis inhibition mechanism in the dental pulp environment; however, the details remain poorly understood.

RANKL expression in dental tissues is observed in odontoblasts, dental pulp cells, periodontal fibroblasts, and odontoclasts^7,8,15–18^. In addition, CSF-1 is expressed in the pulp tissues, and contributes to the proliferation of resident macrophages^19^. These reports suggest that the essential factors for odontoclast differentiation are expressed in the pulp environment. Besides, the dental pulp becomes hypoxic in response to damage or inflammation^20–22^. It is reported that HIF-1α increases and decreases the expression levels of RANKL and OPG in periodontal ligament cells (PDL), respectively^23^. Similarly, RANKL expression in osteocytes is positively regulated by HIF-1α^24^. In addition, HIF-2α upregulates RANKL in osteoblastic cells^25^ or fibroblast-like synoviocytes^26^; however, the effects of traumatic dental tissue damages on odontoclast regulating factors expressed in the pulp environment remain unclear.

A previous study demonstrated the induction of tooth internal resorption in the dental maxillary incisors of pulp-depleted rats, in which the pulp space was replenished with periodontal ligament, alveolar bone cells, or circulating cells^27^. As the researchers observed that dental pulp cells expressed high levels of OPG, they concluded that dental pulp cells negatively regulate odontoclast differentiation via OPG. However, the effect of depleting OPG on odontoclastogenesis in vivo needs to be clarified. It is well known that *OPG-knockout (KO)* mice exhibit severe osteoporosis due to increased osteoclastogenesis in bone tissues^28,29^; however, the phenotypes in healthy or damaged dental pulp tissues of these mice have not been investigated.

In this study, we assessed the potential regulatory mechanism of odontoclastic differentiation in dental pulp in *OPG-KO* mice, and explored the contribution of OPG in the regulation of damage-induced pulpal odontoclastogenesis using a tooth replantation surgery^27^. Our findings provide insights into the requirement of OPG for the maintenance of a steady-state in the normal pulp and the damaged pulp environment.

## Results

### Odontoclast regulatory molecules are expressed in dental pulp environment but anti-differentiation factor OPG is dominant

**Figure 1:**
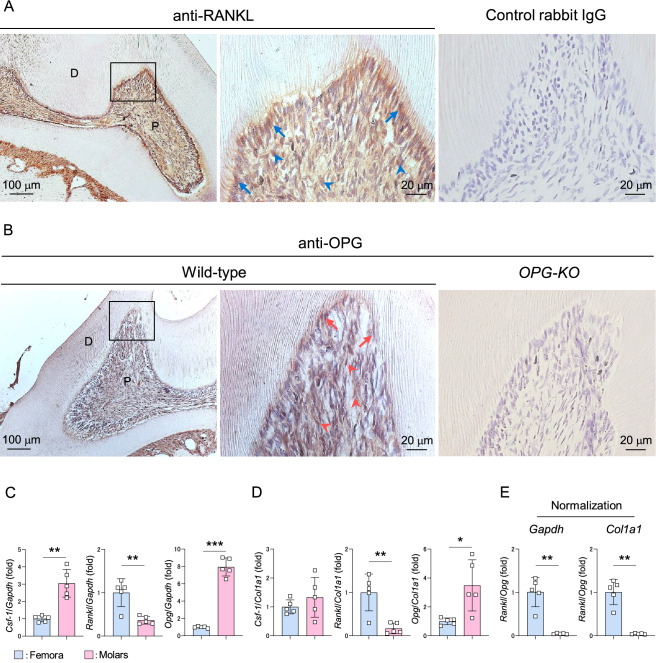
Odontoclast regulatory molecules are expressed but anti-differentiation factor OPG is dominant in the dental pulp environment. (**A**, **B**) Representative images of 6-week-old mouse maxillary first molars stained with anti-RANKL (**A**, left and middle panels) and anti-OPG (**B**, left and middle panels) antibodies. n = 3. Blue arrows: RANKL^+^ odontoblasts, blue arrowheads: RANKL^+^ pulp stromal cells, red arrows: OPG^+^ odontoblasts, red arrowheads: OPG^+^ pulp stromal cells. Normal rabbit IgG (**A**, right panel) and sections from *OPG-KO* mice (**B**, right panel) were used as negative control, respectively. Right panels are magnified views of boxed areas. P: pulp, D: dentin. (**C**–**E**) Real-Time PCR analysis of *Csf-1*, *Rankl,* and *Opg* expression in 6-week-old mouse femora and maxillary first molars normalized with *Gapdh* (**C**) or *Col1a1* (**D**). Relative ratio of *Rankl* to *Opg* normalized with *Gapdh* (left panel) or *Col1a1* (right panel) (**E**). Femora: n = 5, Molars: n = 5. *p < 0.05, **p < 0.01, ***p < 0.001. Data are represented as mean ± SD.

RANKL and OPG were detected in both osteoblasts and osteocytes in mouse femora by immunohistochemical staining (Supplementary Fig. S1). We analyzed the expression pattern of these molecules in mouse dental pulp of maxillary first molars, and detected high expression of RANKL in odontoblasts but modest expression in dental pulp stromal cells (Fig. 1A, blue arrows: RANKL^+^ odontoblasts, blue arrowheads: RANKL^+^ pulp stromal cells). However, similar expression levels of OPG were observed in the entire dental pulp tissue, including odontoblasts and pulp stromal cells (Fig. 1B, red arrows: OPG^+^ odontoblasts, red arrowheads: OPG^+^ pulp stromal cells), but was undetectable in dental pulp tissues from *OPG-KO* mice. Real-Time PCR experiments revealed that the expression levels of *Csf-1* and *Opg* were significantly higher in the mouse maxillary first molars than in the mouse femora (Fig. 1C, left and right panels), with the *Opg* expression consistent with a previous report^27^. Whereas, lower *Rankl* expression was observed in the molars that in the bone tissues (Fig. 1C, middle panel). Unlike molars, RNA obtained from femora are mostly derived from hematopoietic cells, which are not a major provider of osteoclast regulatory factors^5^. To exclude hematopoietic cells, we performed re-normalization of each molecule using the expression of *type 1 collagen alpha 1* (*Col1a1*) mRNA, a tissue-specific marker for osteoblasts or odontoblasts^30^. *Csf-1* levels were comparable between bone and molars; however, the levels of *Rankl* and *Opg* in molars remained significantly lower and higher than those in femora, respectively, in the re-normalized data (Fig. 1D). The relative ratio of *Rankl* to *Opg* in molars was significantly lower than that in femora in both normalized conditions using *glyceraldehyde 3-phosphate dehydrogenase* (*Gapdh*) or *Col1a1* (Fig. 1E). Altogether, these results suggest that the odontoclast inducible factors, CSF-1 and RANKL are detectable in the healthy dental pulp, but high expression of OPG may be a negative regulator of odontoclastogenesis.

### No phenotypic changes under healthy conditions in the dental pulp environment of *OPG-KO* mice

**Figure 2:**
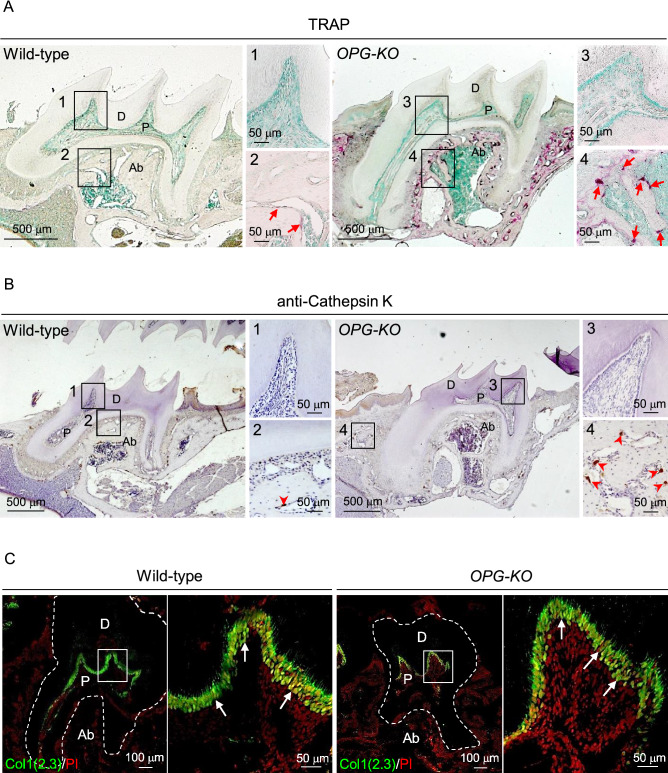
OPG is not required to maintain the pulp environment in normal tooth. (**A**, **B**) Representative images of 6-week-old mouse maxillary first molars from wild-type and *OPG-KO* mice stained with TRAP (**A**) and anti-cathepsin K antibody (**B**). The tissues were counter stained with methyl green (**A**) or hematoxylin (**B**). Red arrows: TRAP^+^ osteoclasts, red arrowheads: cathepsin K^+^ osteoclasts. Numbered panels are magnified views of boxed areas. n = 3. (**C**) Representative confocal images of maxillary first molars from 6-week-old *Col1(2.3)-GFP*; *wild-type* and *Col1(2.3)-GFP*; *OPG-KO* mice. Nuclei were visualized using PI. White arrows: Col1(2.3)-GFP ^+^odontoblasts. Right panels are magnified views of boxed areas. n = 3. P: pulp, D: dentin, Ab: alveolar bone.

To evaluate whether OPG expression is indispensable for pulp environment in the healthy state, the presence of odontoclasts in maxillary first molars of *OPG-KO* mice were analyzed. TRAP and anti-cathepsin K staining revealed that the number of osteoclasts localized in the alveolar bone tissue were higher in *OPG-KO* mice than those in wild-type mice (Fig. 2A,B, red arrows: TRAP^+^ osteoclasts, red arrowheads: cathepsin K^+^ osteoclasts). However, odontoclasts were not observed in dental pulp tissues of both wild-type and *OPG-KO* mice (Fig. 2A,B, squares 1 and 3). In addition, RANKL expression levels were comparable between wild-type and *OPG-KO* mice indicating abundant availability of RANKL for odontoclastogenesis in the *OPG-KO* pulp environment (Supplementary Fig. S2).

The number of osteoblasts reportedly increase in the bone tissue of *OPG-KO* mice due to accelerated bone remodeling^31^. Thus, we next analyzed the effect of *OPG* deficiency on odontoblasts using *Col1(2.3)-GFP* mice, which express odontoblast-specific GFP under the control of a 2.3-kb Col1 promoter fragment^32^. We confirmed the specific expression of GFP in odontoblasts, and detected a comparable distribution pattern of odontoblasts between wild-type and *OPG-KO* mice (Fig. 2C, white arrows: Col1(2.3)-GFP^+^ odontoblasts). These results indicate that high OPG expression is not a reason for the absence of odontoclasts in the healthy pulp tissue, and is dispensable for maintaining a steady-state of odontoblasts.

### Traumatic injury-induced odontoclastogenesis is negatively regulated by OPG in dental pulp environment

**Figure 3:**
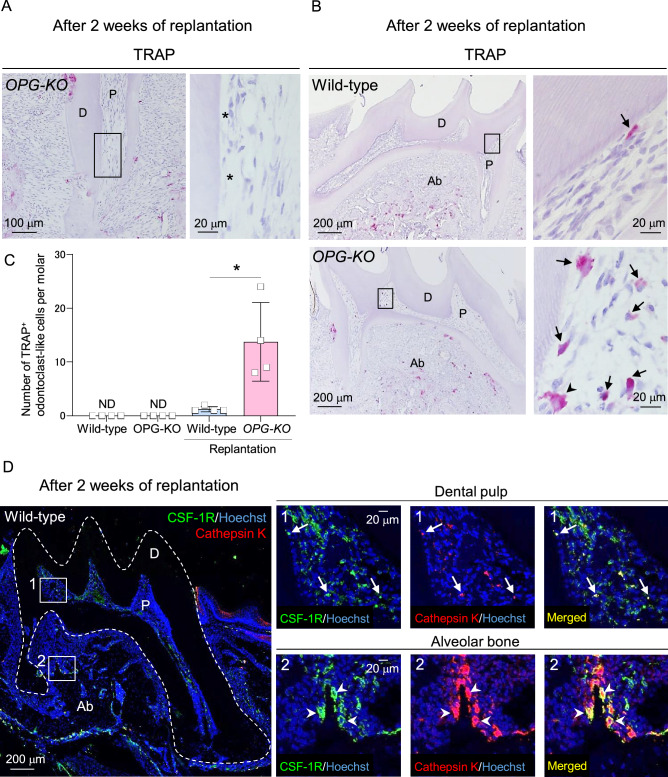
OPG negatively regulates traumatic damage-induced odontoclast differentiation in the dental pulp. (**A**–**C**) Representative images of 8-week-old mice replanted maxillary first molars from wild-type (**B**, upper panels) and *OPG-KO* (**A** and **B**, lower panels) stained with TRAP and hematoxylin. Right panels are magnified views of boxed areas. Asterisks: dentin tissue without odontoblast layer and predentin, black arrows: mono-nucleated TRAP^+^ odontoclast-like cells, black arrowheads: multi-nucleated TRAP^+^ odontoclast-like cells. Quantification of the number of TRAP^+^ odontoclast-like cells in the whole pulp tissue of undamaged and replanted maxillary first molars (**C**). *p < 0.05. Data are represented as mean ± SD; 8-week-old mice; n = 4. (**D**) Representative z-stack confocal images of replanted maxillary first molars from 8-week-old wild-type mice stained with anti-CSF-1R and anti-cathepsin K antibodies. White arrows: CSF-1R^+^ Cathepsin K^+^ odontoclast-like cells, white arrowheads: CSF-1R^+^ Cathepsin K^+^ osteoclasts. Nuclei were stained using Hoechst. Numbered panels are magnified views of boxed areas. n = 3. P: pulp, D: dentin, Ab: alveolar bone, ND: not detected.

Traumatic damages in rodent molars caused by tooth replantation at the original socket right after extraction leads to depletion of parts of the odontoblast layer, and subsequently induces TRAP^+^ cells in dental pulp tissue^33,34^. However, as these TRAP^+^ cells are not observed as dentin-resorbing cells, they are referred to as odontoclast-like cells^33,34^. Thus, we examined the roles of OPG in damage-induced odontoclast formation using the molar replantation model in *OPG-KO* mice. Consistent with previous findings^33^, loss of odontoblast layer and predentin were observed in response to tooth replantation around the root canal wall of *OPG-KO* mice (Fig. 3A, asterisks), and TRAP^+^ odontoclast-like cells were induced in the dental pulp tissue; though, the odontoclasts were not observed in the undamaged dental tissue (Fig. 3B, upper panels and Fig. 3C). Furthermore, a significantly higher number of TRAP^+^ odontoblast-like cells were observed as mono- or multi-nucleated cells in the damage-induced pulp tissue of *OPG-KO* mice (Fig. 3B, lower panels, mono (black arrows)- or multi (black arrowheads)-nucleated TRAP^+^ odontoclast-like cells), compared to wild-type mice (Fig. 3B, upper panels, black arrows: mono-nucleated TRAP^+^ odontoclast-like cells; quantification shown in Fig. 3C). Although, damage-induced TRAP^+^ odontoclast-like cells were not observed as dentin-resorbing cells, the cells were positive for odontoclastic markers like CSF-1R and Cathepsin K (Fig. 3D, CSF-1R^+^ Cathepsin K^+^ odontoclast-like cells (white arrows) and osteoclasts (white arrowheads)). These results indicate that OPG plays a critical role in anti-odontoclastogenesis in damaged dental pulp tissue.

### Traumatic damages of dental tissue have no significant effect on odontoclast precursors in pulp tissues

**Figure 4:**
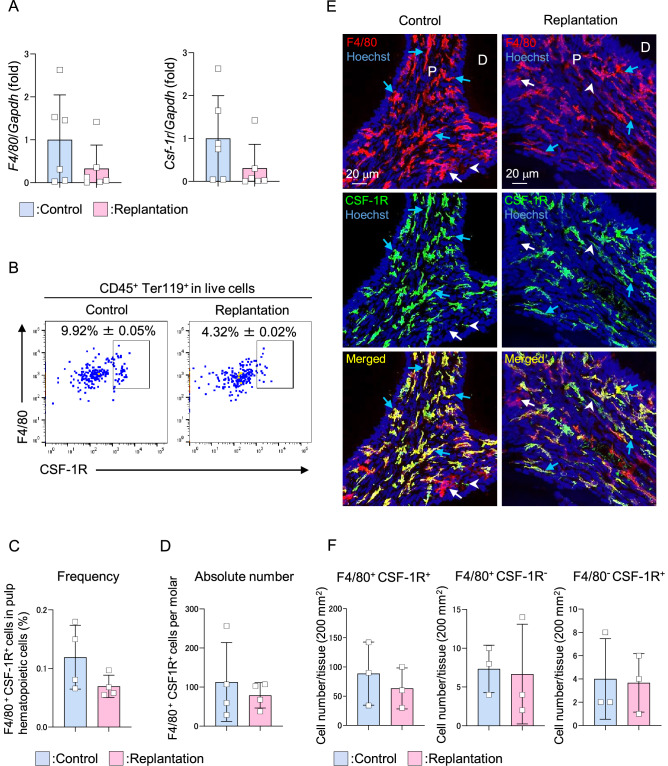
No significant effect on osteoclast precursor in the damaged pulp tissue. Maxillary first molars of 8-week-old wild-type mice were damaged by replantation and used for the experiments 2 weeks later. Another pair of the first molar was used as undamaged control. Real-time PCR analysis for *F4/80* and *Csf-1r* (**A**). n = 6. Representative plots generated by flow cytometry analysis (gated on CD45^+^ Ter119^+^ in live cells) of F4/80^+^ CSF-1R^+^ population in the dental pulp tissue (**B**). Quantification of the frequency (**C**) and absolute number (**D**) of F4/80^+^ CSF-1R^+^ cells in the dental pulp tissue analyzed by flow cytometry. n = 4. Representative confocal images of dental pulp tissue stained with anti-F4/80 and CSF-1R antibodies (**E**). Blue arrows: F4/80^+^ CSF-1R^+^ cells, white arrows: F4/80^+^ CSF-1R^−^ cells, arrowheads: F4/80^−^ CSF-1R^+^ cells. Nuclei were stained using Hoechst. P: pulp, D: dentin. Quantification of F4/80^+^ CSF-1R^+^ cells, F4/80^+^ CSF-1R^−^ cells, and F4/80^−^ CSF-1R^+^ cells were performed using confocal images of dental pulp (**F**). n = 3. Data are represented as mean ± SD.

To delineate the mechanism of induction of odontoclast differentiation in damaged pulp tissue but not in healthy pulp tissue, we analyzed the effects of damages on the pulp macrophages, which are suggested as odontoclasts and osteoclast precursors^1–3,18,35^. The mRNA levels of macrophage markers, *F4/80* and *Csf-1r*, in the damaged molars were not significantly different from healthy control molars (Fig. 4A). A F4/80 and CSF-1R double-positive population was detectable in the molars by flow cytometric analysis (Supplementary Fig. S3). Similarly, the frequency and absolute number of F4/80^+^ CSF-1R^+^ population in dental pulp tissues did not change significantly in response to damages (Fig. 4B–D). Immunofluorescence analysis demonstrated the localization of F4/80^+^ CSF-1R^+^, F4/80^+^ CSF-1R^−^, and F4/80^−^ CSF-1R^+^ cells in healthy pulp tissues (Fig. 4E, blue arrows: F4/80^+^ CSF1R^+^, white arrows: F4/80^+^ CSF-1R^−^, arrowheads: F4/80^−^ CSF-1R^+^ cells). The absolute number of each of the three populations in the damaged dental pulp was comparable with those in the control pulp tissues (Fig. 4F). Altogether, these results suggest that odontoclast precursors include a macrophage population that is not significantly affected by traumatic damages to dental tissues.

### Traumatic damages increase the relative ratio of RANKL/OPG in dental pulp tissue

**Figure 5:**
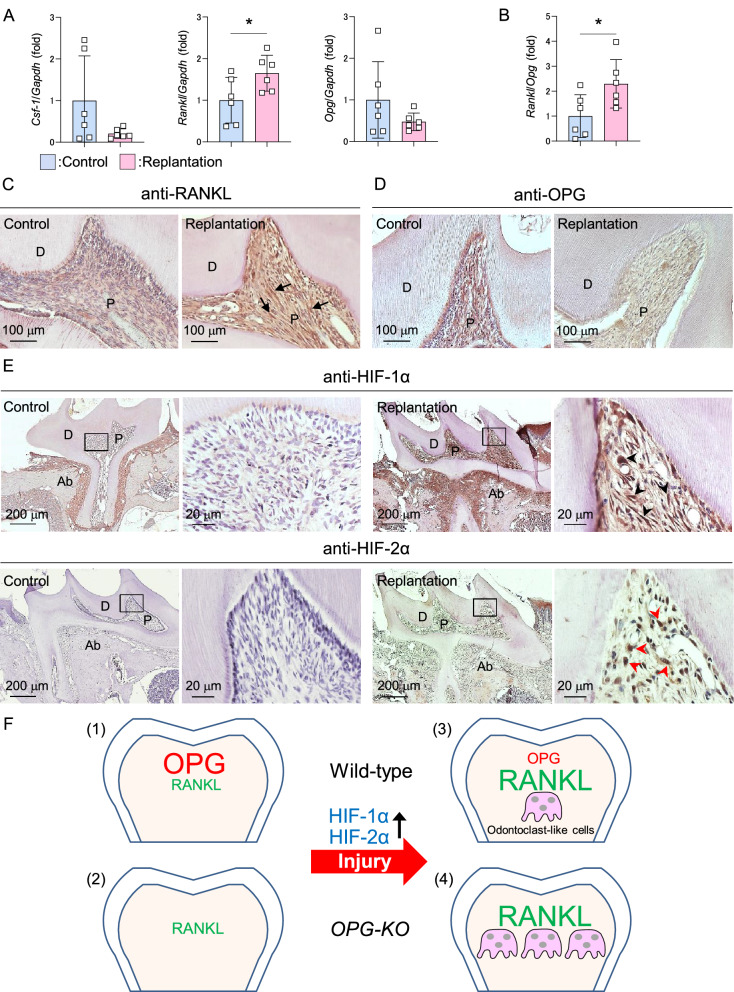
The relative ratio of RANKL/OPG is increased in the damaged dental pulp. (**A**–**E**) Maxillary first molars of 8-week-old wild-type mice were damaged by replantation and used for experiments 2 weeks later. Another pair of the first molar was used as undamaged control. Real-Time PCR analysis for *Csf-1*, *Rankl,* and *Opg* in maxillary first molars normalized with *Gapdh* (**A**). Relative ratio of *Rankl* to *Opg* (**B**). n = 6. *p < 0.05, Data are represented as mean ± SD. Representative images of maxillary first molars from control (**C**, **D**, and **E** left panels) and replantation (**C**, **D**, and **E** right panels) stained with anti-RANKL (**C**), anti-OPG (D), anti-HIF-1α (**E**, upper panels), and anti-HIF-2α (**E**, lower panels) antibodies. n = 5. Arrows: RANKL ^high^ fibroblastic cells, black arrowheads: HIF-1α^+^ fibroblastic cells, red arrowheads: HIF-2α^+^ fibroblastic cells. Right panels are magnified views of boxed areas (**E**). P: pulp, D: dentin, Ab: alveolar bone. (**F**) Schematic representation of regulatory mechanism of odontoclastogenesis in the dental pulp environment. (1) Both RANKL and OPG are expressed in the healthy pulp environment but OPG is dominant. (2) Odontoclasts are not induced in the pulp tissue even when OPG is deleted. (3) Traumatic damages induce odontoclastogenesis via upregulation of relative ratio of RANKL/OPG in the pulp environment. HIF-1α and HIF-2α expression is increased in the damaged pulp tissue. (4) OPG exerts negative regulation for damage-induced odontoclastogenesis in the dental pulp tissue.

Given the lack of effect on odontoclast precursors in damaged molars, we next analyzed the expression levels of odontoblast regulatory molecules in mouse damaged molars using real-time PCR analysis. The expression of *Csf-1* and *Opg* in damaged molars were not significantly different from that in healthy control molars (Fig. 5A, left and right panels). However, *Rankl* expression level was significantly higher in damaged molars than in control molars, resulting in relatively higher levels of *Rankl* compared to *Opg* in the damaged pulp tissue (Fig. 5A, middle panel and Fig. 5B). Immunohistochemical analysis revealed that replantation increased pulpal fibroblastic cells, some of which expressing high level of RANKL (Fig. 5C, arrows: RANKL^high^ fibroblastic cells). In contrast, OPG expression decreased due to replantation (Fig. 5D). Additionally, replantation increased expression of pulpal HIF-1α, reported as a positive and negative regulator of RANKL and OPG, respectively^23,24^ (Fig. 5E upper panels, black arrowheads: HIF-1α^+^ fibroblastic cells). Similarly, the expression of HIF-2α, which increases RANKL expression^25,26^, was also increased in pulpal fibroblastic cells in the damaged dental pulp (Fig. 5E lower panels, red arrowheads: HIF-2α^+^ fibroblastic cells). These results suggest the possibility of odontoclast induction in pulp tissue due to traumatic damages by modulation of expression of odontoclast regulatory factors, RANKL and OPG.

## Discussion

We examined the significance of OPG expression in the normal dental pulp environment using *OPG-KO* mice, and demonstrated that the features of OPG-deficient pulp tissues were comparable to those in wild-type control mice. In contrast, there is an increase in the relative ratio of RANKL/OPG in the dental pulp tissue due to traumatic injury, and OPG is indispensable for pulpal odontoclast suppression in such conditions. In addition, increased HIF-1α and HIF-2α may be a positive regulator of odontoclast differentiation in the damaged pulp tissue (Fig. 5F).

Consistent with a previous report^27^, the relative ratio of RANKL/OPG is significantly lower in normal tooth than in bone tissue where osteoclasts always exist, suggesting that OPG in pulp tissue negatively regulates odontoclastogenesis. However, odontoclasts were not induced, even in the OPG-deficient pulp with dominant RANKL expression. These results indicate that RANKL levels in the normal pulp are not enough for odontoclast induction. Alternatively, normal pulp may not contain odontoclast precursors. Our data indicates that a proportion of pulp population are positive for macrophage markers such as CSF-1 or F4/80. However, pulp cells labeled by F4/80 were previously categorized as dendritic cells^36^. Further studies are needed to clarify the presence or absence of odontoclast precursors in the healthy pulp tissue.

We have previously identified an osteoclast precursor named QOP (quiescent osteoclast precursor), which circulates in the bloodstream and migrates to bone tissue in response to osteotropic stimuli *in vivo*^37,38^. Furthermore, the spleen is suggested as a reservoir of osteoclast precursors, and contributes to osteoclasts through the blood stream during bone tissue damages^39,40^. Based on these findings, we analyzed the contribution of spleen-derived precursors to pulp odontoclast formation using *OPG-KO* mice. However, damage-induced pulp odontoclasts of splenoectomized *OPG-KO* mice were comparable to that of sham operated *OPG-KO* mice (Supplementary Fig. S4). These results suggest that the pulpal odontoclast precursors are supplied not only by the spleen but also by other organs.

We speculated that an increase in the relative ratio of RANKL/OPG might be one of the factors for damage-induced odontoclast differentiation in pulp tissues. We detected increased levels of HIF-1α and HIF-2α in the damaged pulp tissue; however, the manner in which damages modulate odontoclast regulatory factors is still unclear. Osteoclastic bone destruction observed in autoimmune diseases, such as rheumatoid arthritis, is caused by inflammatory cytokines, including IL-1, IL-6, TNF-α, and IL-17, which increase RANKL expression in synovial fibroblasts^4,41,42^. Immunocompetent cells are localized in the dental pulp^43^, and thus the relative ratio of RANKL/OPG may be modified by inflammatory cytokines derived from pulp inflammatory cells in the damaged pulp tissues.

RANKL is expressed by odontoblasts, odontoclasts, and dental pulp cells in the pulp environment, however, the specific cells that contribute to odontoclastogenesis have not yet been identified^7,8,15,16,18,27^. The histological analysis in the present study demonstrates RANKL expression in odontoblasts and pulp stromal cells (Fig. 1A). Since odontoblasts are depleted in response to damage, they are not likely the contributors to odontoclastogenesis (Fig. 3A)^33^. In contrast, human-derived dental pulp stromal cells have the capacity to differentiate into odontoclasts in vitro, suggesting that they may also contribute to odontoclastogenesis *in vivo*^18^. Importantly, there is an increase in fibroblastic RANKL^high^ cells in the damaged pulp environment, suggesting that these cells contribute to damage-induced odontoclast differentiation (Fig. 5C). It will be important for future studies to identify a niche dental pulp environment for odontoclast formation and clarify potential regulatory mechanisms.

In summary, our study demonstrates that the relative ratio of RANKL/OPG is a key factor for damage-induced pulpal odontoclastogenesis, however, pulp cells providing these molecules and their regulatory mechanisms need to be resolved. The significance of RANKL/OPG ratio in odontoclast regulation in the human dental pulp environment also needs to be clarified. Regardless, our data highlights the previously unknown role of pulpal OPG in the negative regulation of odontoclastogenesis in damaged dental pulp, not indicated in healthy conditions.

## Methods

### Experimental animals

C57BL/6 and *OPG-KO*^29^ mice were purchased from Sankyo Labo Service Corporation (Tokyo, Japan) and CLEA Japan (Tokyo, Japan), respectively. *Col1(2.3)-GFP* mice were provided by K. Matsuo (Keio University, Tokyo, Japan)^32^. To generate *Col1(2.3)-GFP*+*/−*; *OPG*+*/−*mice, we crossed *Col1(2.3)-GFP*+*/−*with OPG-KO. Further, the *Col1(2.3)-GFP*+*/−*; *OPG*+*/−and OPG*+*/−*mice were crossed to generate *Col1(2.3)-GFP*+*/−*; *wild-type* mice *and Col1(2.3)-GFP*+*/−*; *OPG-KO* mice. Euthanasia was performed by cervical spine fracture dislocation under anesthesia with isoflurane (Pfizer Inc., New York City, NY, USA), when the mice were used for experiments. All animals were maintained under specific pathogen-free conditions at 24 ± 2 °C and 50–60% humidity with a 12 h light/dark cycle, and were provided with sterilized water and ad libitum diets in the animal facilities certified by the Animal Care and Use Committees of Tokyo Dental College or Matsumoto Dental University. Animal studies were approved and performed in accordance with the guidelines of Tokyo Dental College and Matsumoto Dental University Animal Care Committee.

### Antibodies and reagents

The following primary antibodies were used: rabbit anti-RANKL antibody and rabbit anti-OPG antibody (Bioss Antibodies, Boston, MA, USA), sheep anti-CSF-1R antibody (R & D systems, Minneapolis, MN, USA), rabbit anti-cathepsin K antibody (Abcam, Cambridge, United Kingdom), rat anti-CSF-1R antibody coupled to phycoerythrin (PE) (AFS98), rat anti-CD45 antibody coupled to allophycocyanine (APC) (30-F 11), and rat anti-Ter119 (TER-119) antibody coupled to APC (Thermo Fisher Scientific, Waltham, MA, USA), rat anti-F4/80 antibody coupled to FITC (BM8) and rat anti-F4/80 antibody coupled to PE (BM8) (BioLegend, San Diego, CA, USA), rabbit anti-HIF-1α antibody (GeneTex, Irvine, CA, USA), and rabbit anti-HIF-2α antibody (Novus Biologicals, Centennial, CO, USA).

The following secondary antibodies were used: donkey anti-sheep coupled to Alexa Fluor (AF) 488 (Thermo Fisher Scientific) and goat anti-rabbit coupled to AF Plus 555 (BD Biosciences, San Jose, CA, USA). Nuclei were stained using Hoechst 33342 (Thermo Fisher Scientific) or Propidium iodide (PI) (BD Biosciences).

### Microscopy imaging analysis

To make paraffin-embedded molars or femora, mice were perfused with 4% paraformaldehyde (PFA), and the collected tissues were further fixed with PFA for 24 h at 4 °C. The tissues were decalcified with 10% EDTA for 3 weeks at 4 °C, followed by the use of 4 µm thick sections for TRAP^44^ or immunohistochemical staining. The sections were incubated with primary antibodies for 1 h at 24 ± 2 °C for RANKL and OPG staining, or overnight at 4 °C for cathepsin K staining. Immunoreactivity was visualized using Histofine Simple Stain Mouse MAX PO (R) or Histofine Simple Stain Mouse MAX PO (G) (Nichirei Biosciences, Tokyo Japan) and ImmPACT DAB Substrate (Vector Laboratories, Burlingame, CA, USA). Bright-field images were acquired using an Axioskop2 plus equipped with Plan-NEOFLUAR (2.5×/0.075, 5×/0.16, 10×/0.30, 20×/0.50, 40×/0.75) (Carl Zeiss, Oberkochen, Germany) or by an Axiophot2 with Plan-APOCHROMAT (10×/0.45, 20×/0.75) (Carl Zeiss), and both microscopes were equipped with the AxioVision Rel. 4.8 software (Carl Zeiss).

To prepare cryosections, mice were perfused with 20% formalin, the maxilla was collected, and further fixed with 20% formalin for 8 h at 24 ± 2 °C. The dissected maxilla was decalcified by 20% Morse solution (FUJIFILM Wako Pure Chemical, Osaka, Japan) for 24 h at 4 °C and subsequently incubated in 10%, 20%, and 30% sucrose solutions at 4 °C for more than 2 h; and embedded in super cryo-embedding medium (SCEM) (SECTION-LAB, Hiroshima, Japan). Cryosections of 10 µm thickness were obtained according to the Kawamoto’s film method (Kawamoto and Shimizu, 2000) using Cryofilm Type IIIC (16UF) and tungsten carbide knife (SL-T35UF) (SECTION-LAB). The sections were pretreated with 0.1% Triton X-100 for 10 min, and incubated with primary antibodies overnight at 24 ± 2 °C. The sections were further incubated with secondary antibodies for 2 h at 24 ± 2 °C. Fluorescence images were acquired using laser-scanning confocal microscopes (LSM 510 and LSM 880, Carl Zeiss), equipped with Plan-APOCHROMAT (10×/0.45, 20×/0.8) and ZEN 2.3 black edition (Carl Zeiss). Z-stack confocal projection images were captured at 1 µm intervals of the 10 µm-thick sections.

### Preparation of mouse molars for analysis

Maxillary first molars were extracted from the euthanized mice using hooked forceps. The molars were incubated with HBSS containing 0.1% collagenase IV, 0.2% Dispase (Thermo Fisher Scientific), and 20 U/mL DNase (Worthington Biochemical, Lakewood, NJ; USA) for 15 min at 37 °C to remove periodontal ligaments. The molars were used for flow cytometric and real-time PCR analysis.

### Flow cytometric analysis

Mouse maxillary first molars incubated with HBSS (Thermo Fisher Scientific) and DNase (Worthington Biochemical) as mentioned above, were crushed using forceps and incubated for 30 min at 37 °C to dissociate. The cells were stained with antibodies, and flow cytometric experiments were performed using a FACSMelody equipped with FACSChorus software (BD Biosciences). The data were analyzed with the FlowJo V10 software (BD Biosciences), and dead cells and debris were excluded by FSC, SSC, and PI staining profiles.

### RNA isolation and quantitative real-time PCR

Mouse femora or molars were homogenized in TRIzol reagent (Thermo Fisher Scientific) using Tissue Lyser (Qiagen, Hilden, Germany), and total RNA was purified using the PureLink RNA Micro kit (Thermo Fisher Scientific). Quantitative Real-Time PCR was performed by using the One Step SYBR Prime Script PLUS RT-PCR (TAKARA, Shiga, Japan) using StepOnePlus or 7500 Fast systems (Thermo Fisher Scientific). Gene expression data were normalized to *Gapdh* or *Col1a1* expression. The primers for each gene are shown in Table 1.

**Table 1:** Primers used for Real-time PCR.

Gene	Forward (5′–3′)	Reverse (5′–3′)
*Csf-1*	GAACAGCCTGTCCCATCCATC	TGAGGCCAGCTCAGTGCAA
*Rankl*	CATGTGCCACTGAGAACCTTGAA	CAGGTCCCAGCGCAATGTAAC
*Opg*	CATGAGGTTCCTGCACAGCTTC	ACAGCCCAGTGACCATTCCTAGTTA
*F4/80*	GAGATTGTGGAAGCATCCGAGAC	GACTGTACCCACATGGCTGATGA
*Csf-1r*	GGCCCAGCCTGTATTTGCAC	ACCGCTGCTTGGCAGGTTAG
*Col1a1*	TCAGTGCAATTGTGTTGCTGAAAG	GATACCAAACTGGGCGTGCTG
*Gapdh*	TGTGTCCGTCGTGGATCTGA	TTGCTGTTGAAGTCGCAGGAG

### Tooth replantation model

To induce root damages, mouse maxillary first molars were extracted using hooked forceps under anesthesia with isoflurane (Pfizer) inhaled by a vaporizer (Univentor, Zejtun, Malta), and retransplanted in the original socket immediately. Two weeks later, the maxillary first molars were collected and used for analyses.

### Splenectomy

*OPG-KO* mice were anaesthetized with isoflurane (Pfizer) using a vaporizer (Univentor). The spleen was identified after a transverse laparotomy incision just to the left of the spinal cord and removed after blood vessel ligation. Sham-operated animals underwent the laparotomy without a splenectomy.

### Statistics

Statistical analyses were performed using GraphPad Prism 8 (GraphPad Software, La Jolla, CA, USA). The data were first analyzed with the Shapiro–Wilk test to evaluate normal distribution. To compare two groups, equality of the two variances was assessed using an F-test. Student’s *t*-test was used to assess statistical significance in data sets that met both the test requirements for distribution and variance. Non-parametric Mann–Whitney U-test was used to test data that did not show a normal distribution. Welch’s *t*-test was used to analyze data with significantly different variances in F-test. The results were expressed as mean ± standard deviation (SD), and a *p* < 0.05 was considered as statistically significant.

### Ethical statement

All experiments were carried out in compliance with the ARRIVE guidelines and Guidelines of the Tokyo Dental College Animal Care Committee.

## Supplementary Information

Supplementary Information

## Data Availability

We agree to make available materials, data and associated protocols used in this study upon request.
