# Supplementary material for: RANKL/OPG ratio regulates odontoclastogenesis in damaged dental pulp
Source: Sci Rep. 2021 Feb 25;11:4575. doi: 10.1038/s41598-021-84354-y (PMC7907144; doi:10.1038/s41598-021-84354-y)
Supplement: Supplementary file 1 — Supplementary Information [file 41598_2021_84354_MOESM1_ESM.pdf]

RANKL/OPG Ratio Regulates Odontoclastogenesis in Damaged Dental Pulp

Daisuke Nishida[1], Atsushi Arai[2], Lijuan Zhao[3], Mengyu Yang[3], Yuko Nakamichi[3], Kanji Horibe[4], Akihiro Hosoya[5], Yasuhiro Kobayashi[3], Nobuyuki Udagawa[6,*], Toshihide Mizoguchi[1,6,*]

[1]Oral Health Science Center, Tokyo Dental College, Tokyo 101-0061, Japan

[2]Department of Orthodontics, Matsumoto Dental University, Nagano 399-0781, Japan

[3]Institute for Oral Science, Matsumoto Dental University, Nagano 399-0781, Japan

[4]Department of Oral Histology, Matsumoto Dental University, Nagano 399-0781, Japan

[5]Department of Histology, School of Dentistry, Health Sciences University of Hokkaido, Hokkaido 061-0293, Japan

[6]Department of Oral Biochemistry, Matsumoto Dental University, Nagano 399-0781, Japan

***Corresponding Authors**:

Toshihide Mizoguchi, PhD, Oral Health Science Center, Tokyo Dental College, Tokyo 101-0061, Japan. E-mail: tmizoguchi@tdc.ac.jp

Nobuyuki Udagawa, DDS, PhD, Department of Oral Biochemistry, Matsumoto Dental University, Nagano 399-0781, Japan. E-mail: nobuyuki.udagawa@mdu.ac.jp

Supplementary Information

Supplementary Figure S1

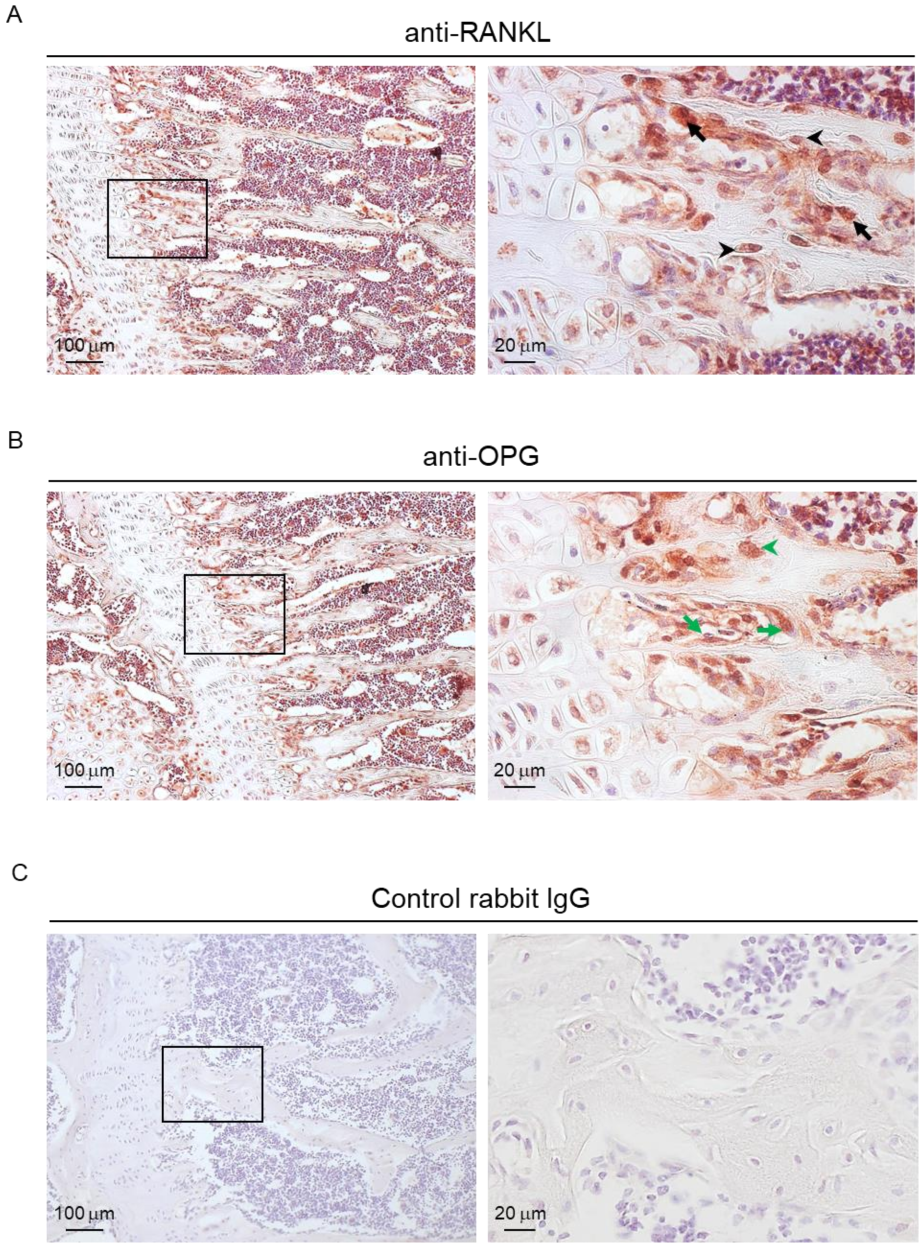

Supplementary Figure S1. RANKL and OPG are expressed by osteoblasts and osteocytes in the bone tissue.

Representative images of 6-week-old mouse femora stained with anti-RANKL (A) and anti-OPG (B) antibodies. n = 3. Black arrows: $RANKL^+$ osteoblasts, black arrowheads: $RANKL^+$ osteocytes, green arrows: OPG^+ osteoblasts, green arrowheads: OPG^+ osteocytes. Normal rabbit IgG were used for negative control (C).

Supplementary Figure S2

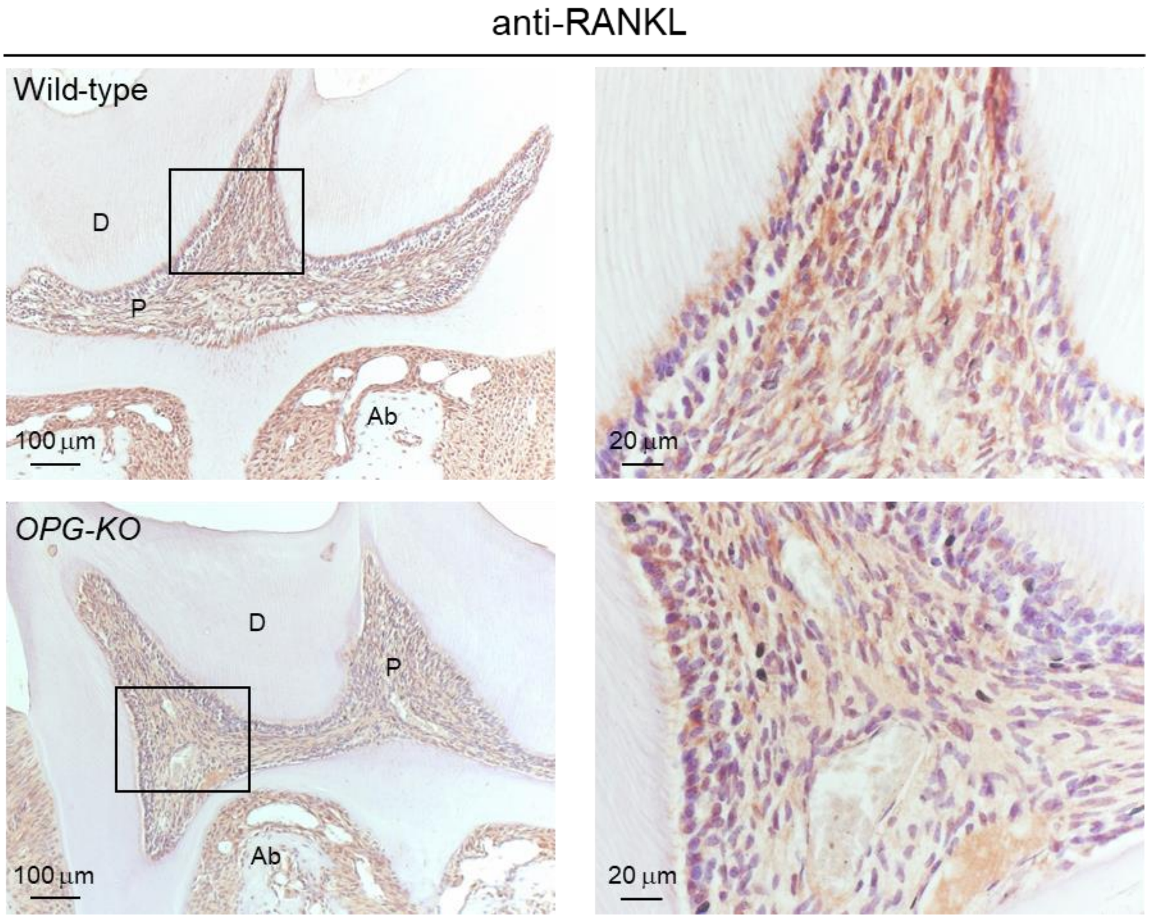

Supplementary Figure S2. RANKL expression level in dental pulp cells is comparable between wild-type and OPG-KO mice.

Representative images of 6-week-old mouse maxillary first molars from wild-type (upper panels) and OPG-KO (lower panels) stained with anti-RANKL antibody. Right panels are magnified views of boxed areas. n = 5. P: pulp, D: dentin, Ab: alveolar bone.

Supplementary Figure S3

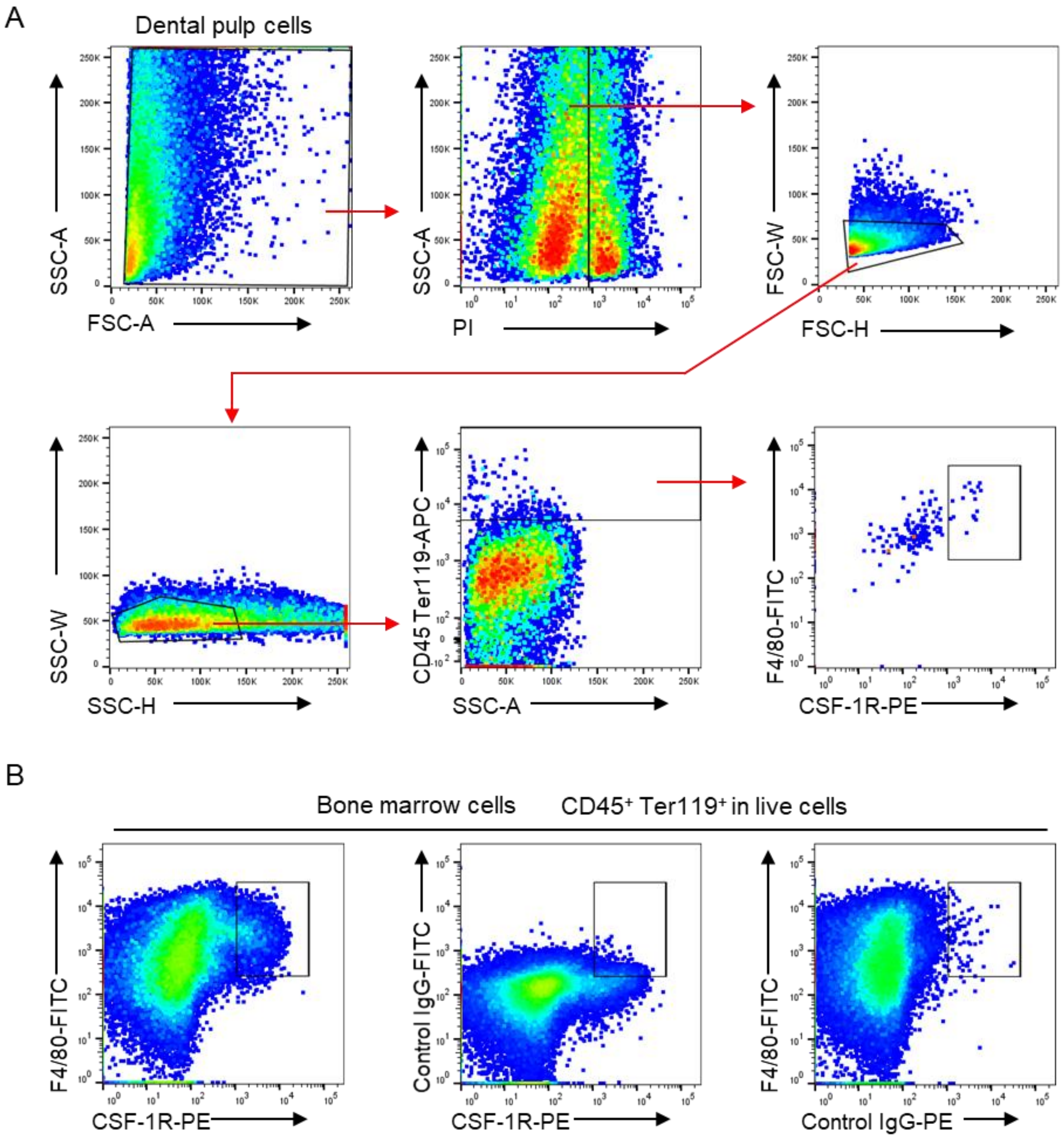

Supplementary Figure S3. Gating strategy for F4/80$^+$ CSF-1R$^+$ cells in the dental pulp tissue

(A) Gating strategy of the flow cytometry for F4/80$^+$ CSF-1R$^+$ cells in the dental pulp tissue from 8-week-old mice. (B) Dot plots generated by flow cytometry (gated on CD45$^+$ Ter119$^+$ in live cells) for F4/80$^+$ CSF-1R$^+$ cells in bone marrow cells from 8-week-old mice (left panel). Negative control IgG conjugated with FITC (middle panel) or with PE (right panel).

Supplementary Figure 4

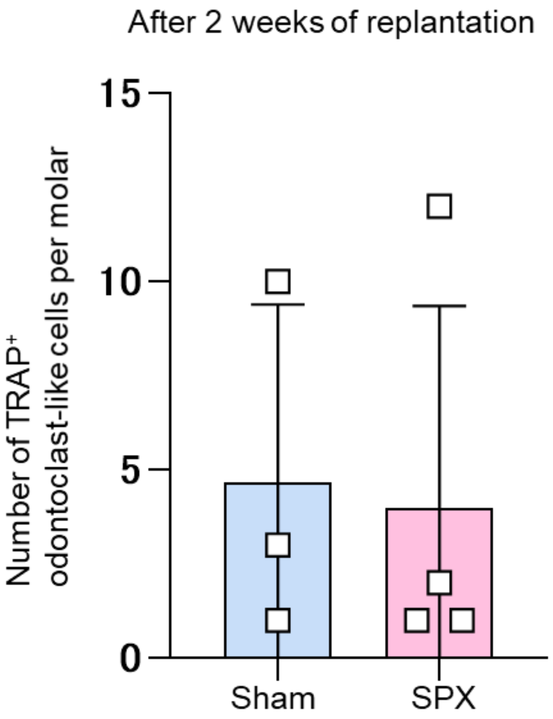

Supplementary Figure S4. Number of damage-induced dental pulp odontoclasts were not changed by splenectomy.

Maxillary first molars of 5-week-old OPG-KO mice were replanted 1 week after splenectomy (SPX). Damage induced odontoclasts in the maxillary first molars were detected by TRAP-staining after 2 weeks replantation, and quantified. Number of odontoclast in the damaged pulp of SPX mice was comparable with sham-operated mice. n = sham: 3, splenectomy: 4.
